# Clinical significance of macrophage phenotypes in cardiovascular disease

**DOI:** 10.1186/s40169-014-0042-1

**Published:** 2014-11-21

**Authors:** Heather J Medbury, Helen Williams, John P Fletcher

**Affiliations:** Vascular Biology Research Centre, Department of Surgery, University of Sydney, Westmead Hospital, Westmead, NSW Australia

**Keywords:** Macrophage, Cardiovascular disease, Atherosclerosis, Plaque stability, M1, M2, Review

## Abstract

The emerging understanding of macrophage subsets and their functions in the atherosclerotic plaque has led to the consensus that M1 macrophages are pro-atherogenic while M2 macrophages may promote plaque stability, primarily though their tissue repair and anti-inflammatory properties. As such, modulating macrophage function to promote plaque stability is an exciting therapeutic prospect. This review will outline the involvement of the different macrophage subsets throughout atherosclerosis progression and in models of regression. It is evident that much of our understanding of macrophage function comes from *in vitro* or small animal models and, while such knowledge is valuable, we have much to learn about the roles of the macrophage subsets in the clinical setting in order to identify the key pathways to target to possibly promote plaque stability.

**Electronic supplementary material:**

The online version of this article (doi:10.1186/s40169-014-0042-1) contains supplementary material, which is available to authorized users.

## Introduction

The main cause of cardiovascular disease is the formation of atherosclerotic plaques within the blood vessel wall. They may occur at multiple sites in the arterial tree and be at different stages of progression [1]. While plaques progressively narrow the arteries in which they form, their clinical significance is dependent more on their composition than the size they attain [2],[3]. Morphologically, advanced plaques are composed of a necrotic core and overlying fibrous cap and those with a relatively large core and thin cap are considered unstable as they are vulnerable to rupture [2]-[4]. Rupture of the cap leads to exposure of the blood to thrombogenic material. While the subsequent thrombus that forms primarily leads to subclinical plaque progression, through fibrosis tissue formation and constrictive remodelling [1],[5], it may also lead to vessel occlusion and occurrence of a clinical event, such as a heart attack or stroke [6],[7].

Macrophages are key players in atherosclerotic plaque development, progression and, importantly, stability as they contribute to formation of the core and degradation of the fibrous cap. However, macrophages can adopt various phenotypes including a wound healing form [8] and, indeed, collagen producing macrophages are present in human carotid plaques [9]. The ‘plasticity’ of macrophages gives hope to the notion of atherosclerotic plaque stabilisation through the modulation of macrophage functions. This review will summarise macrophage phenotype heterogeneity, the presence of the different ‘subsets’ within the plaque throughout its development and focus, in particular, on the possible clinical significance of macrophage subsets in terms of their likely contribution to plaque stability – such as their role in the core and cap.

## Review

### Macrophage phenotypes

Monocytes can differentiate into a spectrum of functional macrophage phenotypes depending upon the microenvironment - such as presence of specific growth and differentiation factors- as well as on the receptors they express, signaling pathways and transcription factors [8],[10]. The first stage of differentiation is induced by macrophage colony stimulating factor (M-CSF) or granulocyte macrophage colony stimulating factor (GM-CSF) [11] and the subsequent phenotype that macrophages adopt is dependent upon the concentration of various mediators they are exposed to - with interferon (IFN)γ and interleukin (IL)-4 priming macrophages to adopt classical or alternative phenotypes respectively [10]. Macrophages exhibit a high degree of plasticity such that some (though not all) of their properties alter as the local milieu changes [12]-[14].

Our understanding of macrophage phenotypes, and their plasticity, relies heavily on cell culture systems and, accordingly, so does much of the terminology applied to them. While numerous names have been used in the literature, the terms that predominate are M1 (classically activated) and M2 (alternatively activated) [15] and as such, these are used here. The M1 and M2 terms parallel the T helper cell (Th)1 and Th2 cytokines which drive macrophage polarisation [16]-[19]. For a review on alternative nomenclature including differentiating based on activation method, the reader is directed to Murray et. al. [20].

M1 macrophages are promoted by Th1 cytokines [15], with this term used in the literature to describe macrophages induced by monocyte stimulation with GM-CSF [21],[22] or by M-CSF combined with lipopolysaccharide (LPS) and IFNγ [23]-[25]. While the cytokine production from both these forms is similar [26], the current recommendation is that GM-CSF macrophages not be assigned the terminology M1 [20]. M1 macrophages are considered inflammatory as they produce high levels of IL-6 and TNFα [27],[28] and they have a recognised role in tissue destruction [8]. M1 macrophages express pro-inflammatory transcription factors such as nuclear factor-κB and signal transducer and activator of transcription (STAT) 1 [28]-[30].

The term ‘M2’ encompasses largely any phenotype that is not M1 [15],[17] and is subdivided into groups based on the stimulus used, with M2a (alternative) stimulated by IL-4 or IL-13, M2b stimulated by immunocomplex and M2c stimulated by IL-10, glucocorticoids or transforming growth factor (TGF)β [15],[31]-[33]. The term ‘M2’ has also been used to describe M-CSF generated macrophages [34] with evidence that M-CSF stimulation promotes expression of a considerable portion of the M2 transcriptome [27]. M2 macrophages (human and mouse) produce anti-inflammatory cytokines such as IL-10 and TGFβ [27],[35]. M2a macrophages express the transcription factors Krüppel-like factor 4, peroxisome proliferator activated receptor-γ (PPARγ) and STAT6 [28]-[30] while M2c macrophages express STAT 3 [36]. The key recognised functions of M2 macrophages are immunosuppressive, including immune regulation and wound healing [8],[15],[35]. There are, in reality, many different modes of activation, resulting in an array of macrophage functional phenotypes [8]. The possible function of these subsets in plaque stability can, in part, be gleaned from understanding the effect of the stimulating cytokines on plaque development in murine models. As such, IFNγ (which promotes M1) is considered pro-atherogenic, IL-4 (which promotes M2a) is considered to have a dual pro and anti-inflammatory character, while IL-10 (which promotes M2c macrophages), is considered anti-atherogenic [37].

In the atherosclerosis field, additional forms have been described including the Mhem macrophage [38] (also known M(Hb) [39] or HA-Mac [40]). Consistent with their presence in regions of haemorrhage, Mhem macrophages arise from culturing monocytes with the haemoglobin/haptoglobin complex [38]-[40]. The term Mox macrophages has been given to murine macrophages (including M1 or M2) cultured in the presence of oxidised phospholipids [41]; their phenotype is markedly different from standard M1 or M2 macrophages. The term ‘M4’ describes macrophages formed when monocytes are differentiated with the platelet chemokine chemokine (C-X-C motif) ligand 4 (CXCL4) [42]. Other monocyte derived cells (sharing some overlapping functions with macrophages) are also recognised in the plaque, such as dendritic cells [43],[44] and fibrocytes [45]. Common markers used to identify the macrophage subsets include CD86 for M1 (as well as Arginase (Arg) II in mice) and CD163 plus CD206 (mannose receptor: MR) for M2 (as well as Arg I and FIZZ1 in mice) [15],[24],[27],[31],[46],[47]. Transcriptome analysis of cultured cells has identified additional markers [27],[28],[48]. Noted differences are, that M2a macrophages also express CD209 [49]; Mhem macrophages, while expressing CD163 and CD206, are distinguishable from M2 macrophages by the expression of activating transcription factor (ATF) [38] and M4 macrophages lack expression of CD163 [50].

### Macrophages in plaque initiation

Atherosclerosis is initiated by the accumulation of apolipoprotien (Apo) B lipoproteins within the vessel wall [51],[52]. Their retention is partially mediated by interaction with extracellular matrix (ECM) proteins, primarily proteoglycans that have chondroitin sulphate side chains [52] such as biglycan and versican [53]-[55]. ECM binding makes lipoproteins susceptible to modification, such as oxidation [56],[57]. This activates endothelial cells (EC) which secrete chemokines that promote monocyte recruitment [58],[59]. Vascular cells, such as EC and smooth muscle cells (SMC), produce M-CSF - a factor which promotes monocyte differentiation into macrophages [60],[61]. The macrophages formed internalise the modified low density lipoprotein (LDL), become foam cells [62] and form what is known as a fatty streak [63]. The inflammatory response to retained lipoproteins is maladapted as the macrophage foam cells do not leave but are retained in the vessel wall [52]. They may also exacerbate lesion formation independently by producing molecules such as lipoprotein lipase [64], sphingomyelinase [65] or proteoglycans [66], which promote lipoprotein retention and modification [67]. Though the contribution of different macrophage subsets to lipoprotein retention is not completely defined, M2a macrophages secrete components of the ECM as part of their wound healing function [8]. Our preliminary findings are that CD163+ foam cells in the plaque produce biglycan (unpublished data) and thus may contribute to retention of lipoproteins.

**Figure 1:**
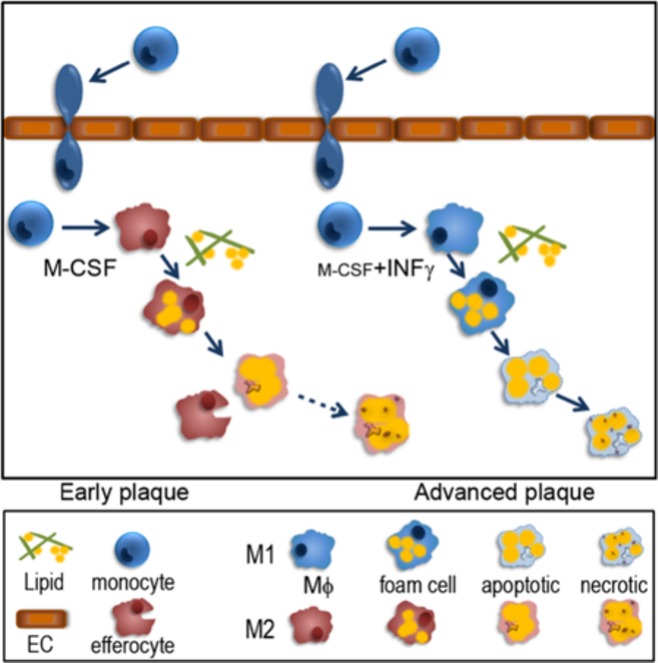
**Proposed role of macrophage subsets in formation of the necrotic core.** Monocytes are recruited early in atherosclerotic plaque development where, through the action of MCSF (and possibly IL-4, as evident in the mouse model), they differentiate into macrophages (Mϕ), primarily skewed towards an M2 form. Through the uptake of modified lipid they become foam cells. Apoptosis of the foam cells is accompanied by efferocytosis, primarily by M2 macrophages. As the plaque adopts an increasingly inflammatory environment, macrophage differentiation skews towards the M1 form and consequently, M1 foam cells predominate. As M1 macrophages have low efferocytosis capability, and there is a decreasing number of M2 efferocytes, apoptotic foam cells (including any remaining M2: dashed line in figure) undergo secondary necrosis promoting development of the necrotic core.

**Figure 2:**
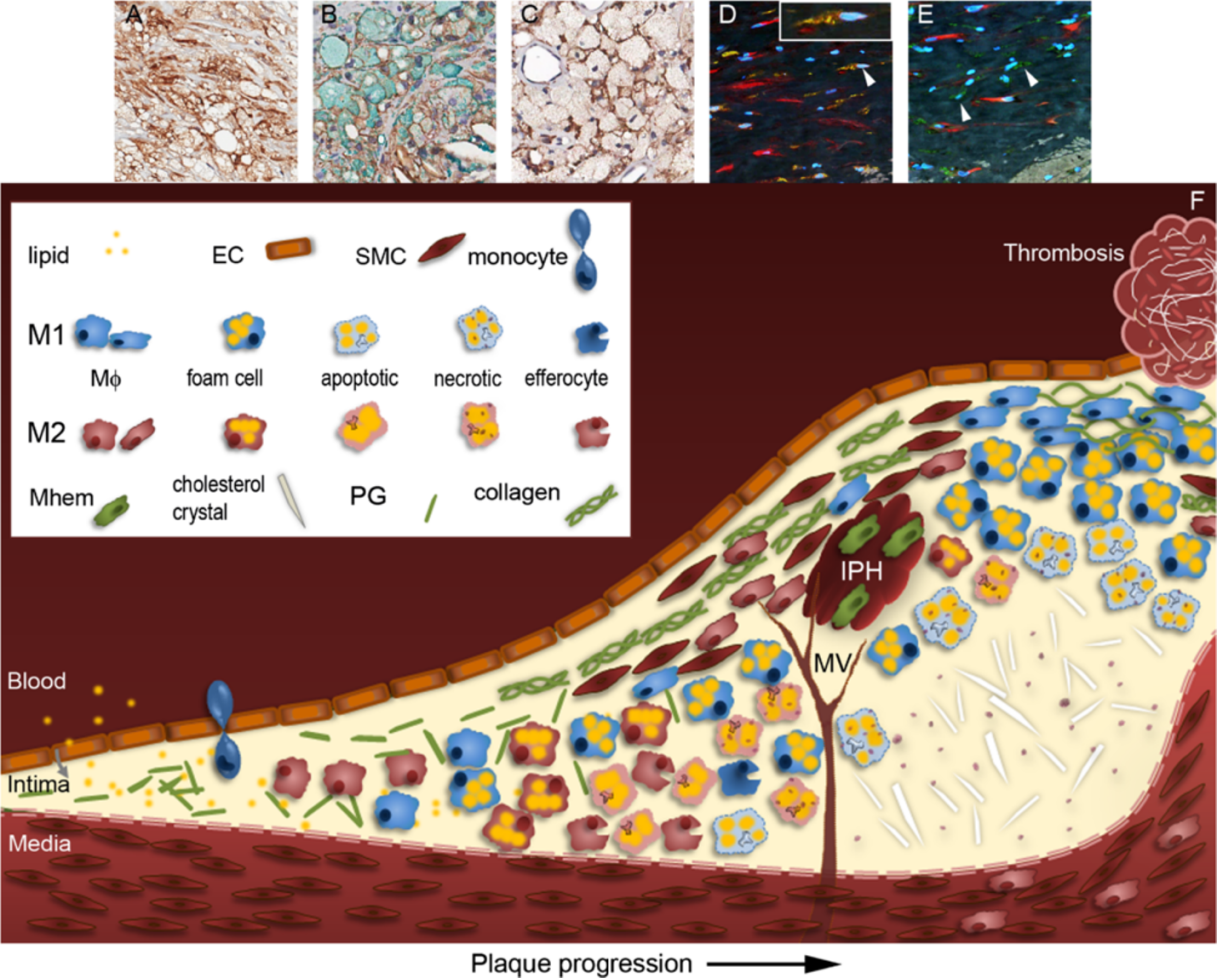
**Macrophages in atherosclerotic plaque development. A**-**C** representative heterogeneous foam cells from carotid atherosclerotic plaques. **A**: CD86 (brown), **B**: CD163 (brown) ADRP (green), **C**: CD206 (brown). **D** and **E**: macrophage collagen I expression. **D**: CD163 (green), procollagen I (red), nuclei (blue), CD163 and procollagen I co-expression (yellow). Inset: closer magnification of the cell in **D** indicated by white arrow head. **E**: CD86 (green: examples indicated by white arrow heads), procollagen I (red), nuclei (blue). Note, no CD86/procollagen I co-expression is evident as seen by absence of yellow. **F**: Proposed role of macrophage involvement in plaque progression. Lipoprotein enters the vessel wall where it is retained, in part, by binding to proteoglycans. Monocytes are recruited into the atherosclerotic plaque where they differentiate into different macrophage phenotypes (predominantly M2 in the early plaque) and uptake modified lipid adopting heterogenous foam cell forms (see also **A**-**C**). Apoptosis of the macrophages, in particular M2, is accompanied by efferocytosis, also primarily by M2 macrophages. As the plaque adopts an increasingly inflammatory environment, M1 foam cells predominate and defective efferocytosis increases, with subsequent necrosis leading to the formation of the necrotic core. In the advanced plaque, intraplaque haemorrhage promotes the formation of Mhem macrophages, which are athero-protective, partly due to reduced lipid accumulation and the production of collagen I. In contrast, M1 macrophages accumulate in the shoulder of the plaque contributing to thinning of the cap through MMP production. The destabilisation of the plaque leads to rupture of the plaque and thrombus formation. IPH = intraplaque haemorrhage, MV = microvessels, Mϕ = macrophage, PG = proteoglycan.

In the murine model (ApoE^-/-^ mouse), it is thought that the early infiltrating macrophages are mainly of the M2 phenotype as they virtually all stain for Arg I [68]. Consistent with this, IL-4 was the predominant transcript (compared to IFNγ) in early lesions [68]. Furthermore, fatty streak formation is significantly reduced in IL-4^-/-^ mice [69]. Whether M2 macrophages predominate in early human plaques is not known, though M-CSF-driven monocyte to macrophage differentiation may promote such skewing (Figure 1). Inferences from the murine model are not entirely appropriate as the initial environment encountered by transmigrating monocytes is quite different to that in humans. There is minimal intima in the mouse [70], while human lesion-prone sites contain considerable diffuse intimal thickening (composed of SMC, elastin and proteoglycans) prior to lipid accumulation, with the lipid depositing deep in the (ECM and SMC rich) intima [71],[72]. In humans, the foam cells form at the interface between infiltrating macrophages and extracellular lipid, rather than just below the luminal surface [62],[73]. As the plaque progresses, a heterogeneous population of foam cells is found (Figure 2:A-C and F), as is evident by the presence of CD68+ foam cells that double stain with a variety of markers such as CD14 (M-CSF derived macrophages (M-Mac)) [74], CD86(M1) [9], CD163(M2) [50] or MR (CD206:M2) [75] .

Conflicting data exists on the ability of different macrophage phenotypes to take up lipid with both increase and decrease of lipid uptake being reported in M2 macrophages- the differences are likely due to variations in culture conditions leading to differences in the cell types being formed and compared. While M2a macrophages take up less lipid than resting macrophages [75], M2 macrophages (a, b and c) take up more lipid than M1 macrophages (M-CSF with LPS plus IFNγ) [76]. M-CSF derived macrophages also take up more lipid than GM-CSF derived macrophages [34]. Macrophages can also take up lipid by non scavenger receptor means such as macropinocytosis [77]; interestingly, this is enhanced in M-CSF plus IL-10 (M2c) compared to GM-CSF derived macrophages [74]. The finding that M2 macrophages take up more lipid than M1 macrophages is consistent with the fact that M-CSF and IL-4 up-regulate the expression of CD36 [34],[78] a receptor for oxLDL [79],[80] and scavenger receptor class A [34],[76] while, conversely, IFNγ reduces CD36 expression [81]. GM-CSF up-regulates expression of genes that promote reverse cholesterol transport (PPARγ, liver x receptor (LXR)-α [34],[74] and ATP-binding cassette sub-family G member 1(ABCG1)) [74]. As M2 (a, b and c) macrophages do not differ in ApoA-1 or high density lipoprotein (HDL)-stimulated cholesterol efflux compared with M1 macrophages, it is thought that the net increase in foam cell formation is primarily due to cholesterol uptake [76]. The accumulation of lipid by M-CSF derived macrophages enhances pro-inflammatory responses characterised by higher production of IL-6, IL-8 and MCP-1 and lower production of IL-10 upon stimulation with LPS [34].

As the atherosclerotic lesion progresses, a pro-inflammatory environment ensues with greater levels of Th1 cytokines (such as IFNγ) compared to Th2 (IL-4) [7]. Consistent with this, lesion progression in the ApoE^-/-^ mouse is associated with an increased prevalence of M1(Arg II) in older mice [68]. Thus, though M2 macrophages may theoretically have a greater ability to take up lipid in the plaque, the increasingly pro-inflammatory environment may skew monocyte to macrophage differentiation towards that of an M1 phenotype. This skewing would accordingly account for the reported absence of M2 foam cells in advanced human lesions [40], or their location distant from the core [75]. Though, interestingly, in the ApoE^-/-^ mouse, M2 (MR+) macrophages were localised more centrally within the plaque, and had a higher proportion of adipose differentiation-related protein (ADRP) expression compared to M1(chemokine (C-C motif) receptor 7 (CCR7)) macrophages [82]. The lack of M2 foam cells may also arise from increased cell death, as cholesterol uptake promotes endoplasmic reticulum (ER) stress which triggers the unfolded protein response [76],[83] and M2 (IL-13 derived) foam cells are more sensitive to the unfolded protein response than other forms of macrophages [84].

### Macrophages and formation of the necrotic core

The clearance of apoptotic cells promotes resolution of inflammation through the production of anti inflammatory mediators such as IL-10 and TGFβ [85]-[87]. However, in atherosclerosis, defective clearance of apoptotic cells leads to secondary necrosis and development of the necrotic core [88],[89]. The switch from an M2 to an M1 promoting environment during atherosclerosis progression may impede apoptotic cell clearance as M2 cells have greater capacity for efferocytosis [90] (Figure 1); this is through various pathways such as the expression of MR [91] and up-regulation of MER proto-oncogene tryosine kinase (MERTK) (on M2c), which is not induced on M1 macrophages [49],[92]. Furthermore, inhibition of autophagy promotes apoptosis and defective efferocytosis leading to increased plaque necrosis in a murine model [93]. Interestingly, ER stress, which promotes autophagy [93], also promotes an M2 macrophage phenotype [76], while mechanistic target of rapamcyin (mTOR) which negatively regulates autophagy [94], also inhibits M2 polarisation [95]. In addition, the uptake of phospholipid (and adoption of a Mox phenotype) reduces the ability of both M1 and M2 macrophages to phagocytose apoptotic cells [41]. Necrosis leads to a pro-inflammatory state, which itself promotes formation of efferocytic low macrophage phenotypes [90].

### Macrophages in the fibrous cap

While a large necrotic core promotes plaque instability, formation of the fibrous cap promotes plaque stability and thus the role of macrophages in the cap is equally important. Both M1 (CD86) and M2 (CD163 and MR) macrophages are found in the atherosclerotic cap, where they adopt a spindle shape (Figure 2: D-F) [9],[96]. A high number of CD68 macrophages in the cap is associated with plaque instability [97],[98], with this association also holding for M1(CD86), but not M2(CD163), macrophages [9]. Similarly, levels of CD68 and CD11c (M1) in the carotid plaque are higher in symptomatic patients compared to asymptomatic patients, while levels of the M2 markers (CD163 and MR) are lower [99]. Notably, M1 macrophages are found in the rupture-prone shoulder regions of the plaque [96]. Macrophage activity in the cap is highly detrimental as they produce matrix metalloproteinases (MMP) which degrade components of the matrix, thinning the cap and leaving it vulnerable to rupture [100]-[102]. That M1 macrophages are more frequent in plaques with an unstable morphology is consistent with the understanding that M1 macrophages are involved in tissue destruction [8]. This can be directly through the production of matrix metalloproteinases and indirectly through effects on SMC. Macrophage production of inflammatory cytokines, such as IL-1 and TNFα, can stimulate SMC to produce gelatinase, interstitial collagenase and stromelysin [103]. Furthermore, TNFα promotes macrophage–induced vascular SMC apoptosis [104], thus reducing the source of collagen and other matrix which thickens the cap. These cytokines also further activate EC and SMC, up-regulating chemokine production [105].

M2 macrophages may promote plaque stability due to their promotion of tissue repair and evidence of this in the carotid plaque is seen by their (CD163+ and CD206+ macrophages) production of collagen I (Figure 2:D) [9]. Despite this function however, no correlation was found in levels of CD163 in plaque cap, with cap thickness [9]; which may reflect a range of macrophages in the plaque that can express CD163. Furthermore, M2 macrophages may also promote plaque stabilisation by inducing the proliferation of vascular SMC [68].

### Macrophages in the complex plaque

Advanced plaques can become quite complex with features such as calcification and intra-plaque haemorrhage. In this respect, distinct macrophages are found in regions of plaque haemorrhage displaying a non foam cell form [38]-[40]. *In vitro* investigation of these Mhem macrophages shows that they are resistant to foam cell formation through down regulation of scavenger receptors and up-regulation of ATP-binding cassette, sub-family A member 1 (ABCA1), ABCG1 [39] and LXR-β [38]. Consistent with this, MR (CD206) + foam cells in the plaque are smaller and contain smaller lipid droplets than their MR- counterparts [75]. Mhem macrophages are thought to be athero-protective as haemoglobin binding to CD163 up-regulates haemoxygenase (HMOX)1 [106]. HMOX1 catabolises haeme, thus removing its pro-oxidative and pro- inflammatory actions, and in the process, promotes anti-oxidant and anti-inflammatory effects through the generation of haeme degradation by-products, such as biliverdin [107]. Over-expression of HMOX1 inhibits atherosclerosis in ApoE^-/-^ mice [108]. With the production of collagen I evident in CD163+ and CD206+ macrophages found in regions of haemorrhage [9], this suggests that Mhem macrophages may also be athero-protective through production of collagen I. M4 macrophages are also evident in the plaque; they may have a pro-atherogenic role as CXCL4 deficiency results in decreased atherosclerotic plaque burden [109]. Furthermore, *in vitro*, CXCL4 down regulates both IL-10 secretion and CD163 expression and inhibits HMOX1 up-regulation [50].

### Macrophage phenotypes in plaque regression/stabilisation

Plaque regression or stabilisation, a key clinical goal, has been achieved in mouse models, most notably in the Reversa mouse – a mouse in which hypercholesterolaemia (due to knock out of the LDL receptor) can be conditionally reversed [110]. Decreasing LDL resulted in stabilisation of the plaque with a reduced lipid component and increased collagen content. These changes were associated with a decrease in total macrophages (CD68 and Moma +) and increased gene expression of M2 markers such as Arg I, MR, CD163, C-lectin and FIZZ1 [111]. This increase in M2 macrophages is also evident in other models of plaque regression including transplant of the atherosclerosed vessel into normal cholesterolaemic mice [112] and induction of regression by HDL [113]. Whether these changes involved a phenotypic conversion of M1 to M2 macrophages is not clear, but it has been suggested to occur in the ApoE^-/-^ mouse as seen by the presence of macrophages double staining with Arg I (M2) and Arg II (M1) [68], though it should be noted that the specificity of Arg I for M2 macrophages is in question [20]. Nonetheless, an M1- M2 switch has been seen in other models, such as wound healing [114].

The polarisation towards an M2 phenotype in plaque regression is consistent with the view that M1 macrophages are pro-atherogenic and promote an unstable plaque, while M2 macrophages promote tissue repair [10] and likely plaque stability. Stimulation of the PPARγ pathway, which promotes M2 macrophage polarisation [115], results in decreased atherosclerosis development in the ApoE^-/-^ mouse [116]. Interestingly, Wolfs et al. [117] observed reduced atherosclerosis in the LDLR^-/-^ mouse after injection of helminth antigens which reprogrammed monocytes and macrophages to an M2 phenotype. Of note, a link between Schistosomal infection and reduced incidence of atherosclerosis has previously been recognised [118]. These results show that modulation towards an M2 phenotype may inhibit plaque progression, *reflect* plaque regression and holds promise that it may also *promote* plaque regression in an advanced plaque.

### Plasticity of macrophage phenotypes

Though the plasticity of macrophages *in vitro* and *in vivo,* which suggests functional adaptivity, has been documented [12]-[14],[119]-[121], the reversal of the phenotype does not always occur and may depend upon the state of macrophage differentiation. For example, while PPARγ activation primes monocytes to adopt an M2 phenotype, it does not influence M2 marker expression in M1 macrophages nor does it influence the expression of M2 markers in human atherosclerotic lesions [115]. Furthermore, while M-CSF and IL-10 promote the formation of an M2c macrophage and accordingly high levels of expression of MERTK and ability to clear apoptotic cells [49], chronic pre-exposure of the cells to IFN-γ or IL-4 prior to exposure with IL-10 down regulates MERTK, leading instead to the cells up-regulating Fas (CD95) and undergoing apoptosis [92]. In addition, M-CSF was unable to significantly induce CD163 expression on monocytes pre-exposed to CXCL4 [50]. Clearly a greater understanding of macrophage function in the plaque, their plasticity (or lack thereof) and the pathways involved is required to ensure that a plaque stabilising form can be promoted.

## Conclusion

A spectrum of macrophage phenotypes is present in the atherosclerotic plaque with each, in some way, impacting plaque stability. Given the association of M1 macrophages with plaque instability and their known role in tissue destruction, decreasing the levels of these macrophages in the plaque is a promising avenue for plaque stabilisation. However, promoting the elevation of M2 macrophages in the plaque is too simplistic and requires a greater understanding of the function of the various subsets within the human plaque and careful consideration of the pathways to target. For while M2 macrophages may have predominantly anti-atherogenic functions, some properties may promote plaque progression; such as their increased uptake of, and sensitivity to, oxLDL, which may promote enlargement of the core. Furthermore, while macrophages are ‘plastic’, it is apparent that such plasticity is quite conditional with some, but not all, properties being reversible and even leading to undesired functions. (Note also that the source of macrophages in the plaque; the contribution of monocyte derived, proliferating and resident macrophages, to plaque stability will also need to be considered, but this was outside the scope of this review).

Upon further investigation, modulating macrophage function to promote plaque stabilisation may become a reality. However, any approach to modulate macrophage phenotype should be an adjunct to existing treatments of lowering lipids, for lipid deposition in the arterial wall is a key initiating factor in atherosclerosis and itself increases the inflammatory nature of the plaque, which could counteract efforts to promote a less inflammatory environment.

## Authors’ original submitted files for images

Below are the links to the authors’ original submitted files for images. Authors’ original file for figure 1 Authors’ original file for figure 2

## Abbreviations

ABCA1: ATP-binding cassette, sub-family A member 1
ABCG1: ATP-binding cassette sub-family G member 1
ADRP: Adipose differentiation-related protein
Apo: Apolipoprotein
Arg: Arginase
ATF: Activating transcription factor
CCR7: Chemokine (C-C motif) receptor 7
CXCL4: Chemokine (C-X-C motif) ligand 4
EC: Endothelial cell
ECM: Extra cellular matrix
ER: Endoplasmic reticulum
GM-CSF: Granulocyte macrophage colony stimulating factor
HDL: High density lipoprotein
HMOX: Haemoxygenase
IL: Interleukin
IFN: Interferon
LDL: Low density lipoprotein
LPS: Lipopolysaccharide
LXR: Liver x receptor
M1: Type 1 macrophage
M2: Type 2 macrophage
M4: CXCL4 derived macrophage
M-Mac: M-CSF derived macrophage
MCP-1: Monocyte chemotactic protein 1
M-CSF: Macrophage colony stimulating factor
MERTK: MER proto-oncogene, tyrosine kinase
Mhem: Haeme directed macrophage
Mox: Oxidised phospholipid derived macrophages
MR: Mannose receptor
MTOR: Mechanistic target of rapamcyin
OxLDL: Oxidised LDL
PPARγ: Peroxisome proliferator-activated receptor gamma
SMC: Smooth muscle cells
STAT: Signal transducer and activator of transcription
TGFβ: Transforming growth factor beta
Th 1: T helper cell type 1 cytokines
TNF: Tumour necrosis factor
