# Supplementary figures and images for: Clinical significance of macrophage phenotypes in cardiovascular disease
Source: Clin Transl Med. 2014 Nov 21;3:63. doi: 10.1186/s40169-014-0042-1 (PMC4303745; doi:10.1186/s40169-014-0042-1)

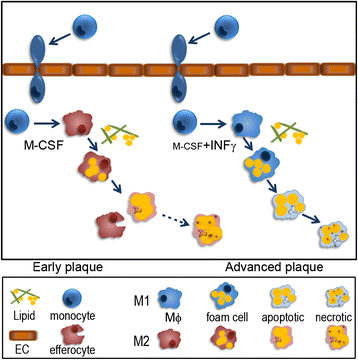

Supplement: Supplementary file 1 — Authors’ original file for figure 1 [file 40169_2014_42_MOESM1_ESM.gif]

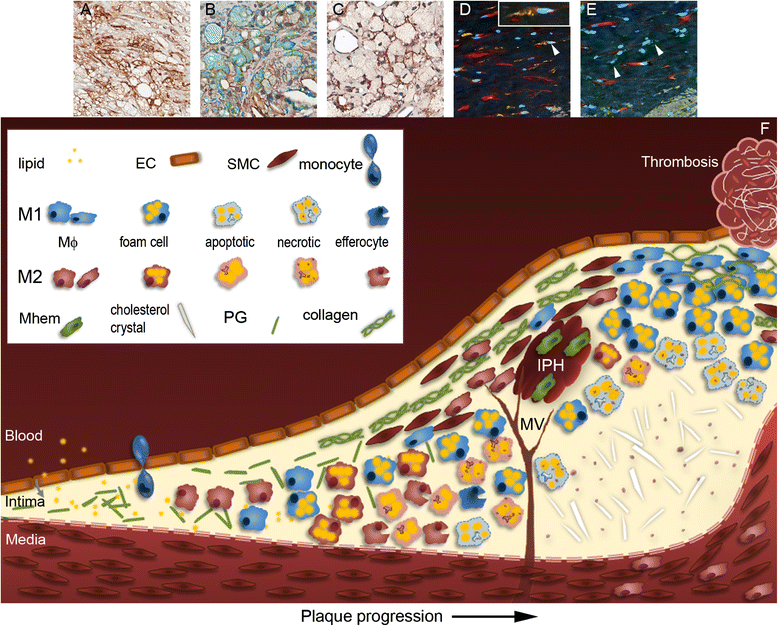

Supplement: Supplementary file 2 — Authors’ original file for figure 2 [file 40169_2014_42_MOESM2_ESM.gif]
